# Multidrug-resistant *Pseudomonas aeruginosa* is predisposed to *lasR* mutation through up-regulated activity of efflux pumps in non-cystic fibrosis bronchiectasis patients

**DOI:** 10.3389/fcimb.2022.934439

**Published:** 2022-07-27

**Authors:** Fengming Ding, Lei Han, Yishu Xue, Iris Tingshiuan Yang, Xinxin Fan, Rong Tang, Chen Zhang, Miao Zhu, Xue Tian, Ping Shao, Min Zhang

**Affiliations:** ^1^ Department of Respiratory and Critical Care Medicine, Shanghai General Hospital, Shanghai Jiao Tong University School of Medicine, Shanghai, China; ^2^ Department of Microbiology, University of Washington, Seattle, WA, United States; ^3^ Department of Tuberculosis, Fuzhou Pulmonary Hospital of Fujian Province, Fuzhou, China; ^4^ Department of Clinical Laboratory, Shanghai General Hospital, Shanghai Jiao Tong University School of Medicine, Shanghai, China; ^5^ Department of Medicine, Dinfectome Inc., Nanjing, China; ^6^ Department of Bioinformatics and System Development, Dinfectome Inc., Nanjing, China

**Keywords:** *Pseudomonas aeruginosa*, multidrug resistance, non-cystic fibrosis bronchiectasis, efflux pump, *LasR* activity

## Abstract

**Background:**

Multidrug-resistant (MDR) *Pseudomonas aeruginosa* is a frequent opportunistic pathogen that causes significant mortality in patients with non-cystic fibrosis bronchiectasis (NCFB). Although the quorum sensing (QS) system is a potential target for treatment, *lasR* mutants that present with a QS-deficient phenotype have been frequently reported among clinical *P. aeruginosa* isolates. We aimed to investigate whether antibiotic resistance would select for *lasR* mutants during chronic *P. aeruginosa* lung infection and determine the mechanism underlying the phenomenon.

**Methods:**

We prospectively evaluated episodes of chronic *P. aeruginosa* lung infections in NCFB patients over a 2-year period at two centers of our institution. QS phenotypic assessments and whole-genome sequencing (WGS) of *P. aeruginosa* isolates were performed. Evolution experiments were conducted to confirm the emergence of *lasR* mutants in clinical MDR *P. aeruginosa* cultures.

**Results:**

We analyzed episodes of *P. aeruginosa* infection among 97 NCFB patients and found only prior carbapenem exposure independently predictive of the isolation of MDR *P. aeruginosa* strains. Compared with non-MDR isolates, MDR isolates presented significantly QS-deficient phenotypes, which could not be complemented by the exogenous addition of 3OC12-HSL. The paired isolates showed that their QS-phenotype deficiency occurred after MDR was developed. Whole-genome sequencing analysis revealed that *lasR* nonsynonymous mutations were significantly more frequent in MDR isolates, and positive correlations of mutation frequencies were observed between genes of *lasR* and negative-efflux-pump regulators (*nalC* and *mexZ*). The addition of the efflux pump inhibitor PAβN could not only promote QS phenotypes of these MDR isolates but also delay the early emergence of *lasR* mutants in evolution experiments.

**Conclusions:**

Our data indicated that MDR *P. aeruginosa* was predisposed to *lasR* mutation through the upregulated activity of efflux pumps. These findings suggest that anti-QS therapy combined with efflux pump inhibitors might be a potential strategy for NCFB patients in the challenge of MDR *P. aeruginosa* infections.

## Introduction

*Pseudomonas aeruginosa* is a frequent opportunistic pathogen that causes chronic infection and releases a number of virulence factors in patients with non-cystic fibrosis bronchiectasis (NCFB), leading to significant morbidity, reduced quality of life, and high treatment burdens (Wilson et al., 2016). Although antipseudomonal agents such as fluoroquinolone, carbapenem, antipseudomonal β-lactam, and penicillin β-lactamase inhibitors are currently recommended as empirical treatment options, eradication of *P. aeruginosa* has become increasingly difficult due to its remarkable capacity to resist antibiotics. The multidrug resistance (MDR) of *P. aeruginosa* can be correlated to variable mechanisms such as overexpression of efflux pumps, synthesis of enzymes, nonresponding modified targets, and biofilm formation (Pang et al., 2019). Among these mechanisms, efflux pumps that pump out the antibacterial drugs from bacterial cells to extracellular environments have received increasing attention, as some antipseudomonal agent exposure can upregulate the activity of efflux pumps, which results in elevated resistance to a broad range of antibiotics (Poole, 2004). The development of MDR can influence the evolution and adaption of *P. aeruginosa* isolates and facilitate their becoming frequent colonizers in patients with chronic infection (Tsukayama et al., 2004). During the process, the function of some physiological systems may be changed in these bacteria, and quorum sensing (QS) is one of such systems.

*P. aeruginosa* uses the QS system to regulate the expression of hundreds of genes in a cell density-dependent manner, and many of these genes code for the production of secreted virulence factors, such as protease, elastase, hydrogen cyanide, and phenazines, which make the QS system components the potential target for antivirulence therapies (Mühlen and Dersch, 2016). QS is mediated by a number of small signal molecules, including N-3-oxo-dodecanoyl-homoserine lactone (3OC12-HSL) and N-butanoyl-homoserine lactone (C4-HSL), which are two key signals that hierarchically activate the LasR-LasI system and the RhlR-RhlI system (Schuster and Greenberg, 2006). 3OC12-HSL is the product of LasI synthase, and when its environmental concentration increases to a threshold, it can bind to the transcription activator LasR and activate a number of genes, including *rhlI*, which codes for the C4-HSL signal synthase, and *rhlR*, which codes for C4-HSL receptor (Whiteley et al., 2017). In addition to these two systems, there is another QS system in *P. aeruginosa* mediated by 2-alkyl-quinolones, which involves the binding of 2-heptyl-3-hydroxy-4-quinolone (*Pseudomonas* quinolone signal (PQS)) or its biosynthetic precursor, 4-hydroxy-2-heptylquinoline (HHQ), to the transcriptional regulator PqsR (Wade et al., 2005).

Although QS is required for full virulence in laboratory *P. aeruginosa* strains, isolates from patients with chronic lung infection are often QS deficient (Heurlier et al., 2006; D'Argenio et al., 2007). Mutant *lasR* strains are the most frequent, whose frequencies have been reported to be greater than 30% in some patients (Smith et al., 2006; Hoffman et al., 2009). The mechanisms underlying this phenomenon have remained poorly understood. It is possible that there is a strong selective pressure against LasR activity so that the *lasR* mutant might have a physiological advantage under certain growth conditions (Heurlier et al., 2005). Alternatively, *lasR* mutants may emerge by social exploitation as they benefit from the extracellular products made by the QS-proficient wild-type strains without paying the metabolic cost (Dandekar et al., 2012). Previous studies reported that *lasR* mutants evolved from a wild-type ancestor during long-term culturing under conditions that required QS and had a selective advantage when cocultured with the wild-type parent (Diggle et al., 2007; Sandoz et al., 2007). In chronic lung infection, such social conflict should be of particular significance as it would result in increased competition and selection for *lasR* mutants (West et al., 2006).

We are interested in understanding whether antibiotic resistance would select for *lasR* mutants during chronic *P. aeruginosa* lung infection. Previous studies suggest there is an interaction between the emergence of MDR and QS function in *P. aeruginosa.* On the one hand, QS contributes to the MDR emergence by regulating the expression of multidrug efflux pump genes (Maseda et al., 2004). On the other hand, the QS system itself is also affected by the expression of multidrug efflux pumps. It is reported that overexpression of multidrug efflux pumps MexCD-OprJ and MexEF-OprJ can shut down the QS response of *P. aeruginosa* through the extrusion of HHQ and kynurenine, which results in the low PQS intracellular levels and reduced production of QS-controlled virulence factors (Lamarche and Déziel, 2011; Olivares et al., 2012; Alcalde-Rico et al., 2018). Efflux pumps, such as MexAB-OprM, have also been proposed to extrude 3-oxo-C12-HSL and other acyl-homoserine lactone compounds (Evans et al., 1998; Pearson et al., 1999; Minagawa et al., 2012). However, so far, whether the impact of efflux pumps on QS would form selection pressure against LasR activity in clinical *P. aeruginosa* strains is not clear.

In this study, we collected *P. aeruginosa* isolates from respiratory tracts in NCFB patients and compared their QS phenotypes between isolates with and without MDR. Whole-genome sequencing (WGS) and evolution experiments were used to characterize the impact of MDR mechanisms on the evolution of *lasR* mutants. This work will further our understanding of the evolution of *P. aeruginosa* QS in chronic lung infection, which would have significant implications for ongoing efforts to develop anti-QS-based therapies against multidrug-resistant *P. aeruginosa* (MDRPA) (Mellbye and Schuster, 2011).

## Materials and methods

### Clinical and microbiological data

We conducted a two-center prospective investigation of chronic *P. aeruginosa* lung infections in adult (age ≥18 years) patients at Shanghai General Hospital (SGH) occurring between 01 August 2019 and 31 July 2021. One center was at the north Hongkou Campus in the urban area, and the other center was at the south Songjiang Campus in the suburban area. The criteria for inclusion in the study were patients diagnosed with confirmed NCFB and having repeated positive results of *P. aeruginosa* cultures from lower respiratory samples such as sputum and bronchoalveolar lavage fluids. The *P. aeruginosa* infection lasted for more than 14 days. Patients co-colonized with other detected pathogens in airways were excluded from the analysis. Clinical information, including age, gender, etiology, duration of infection, association with a hospital-acquired infection, intensive care unit stay, ventilator use, prior antibiotic exposures within 90 days of *P. aeruginosa* isolation, and absolute neutrophil count in peripheral blood, were obtained from the electronic medical record.

The results of antimicrobial susceptibility testing were performed by the SGH microbiology laboratory using VITEK2 (bioMérieux, Durham, NC, USA). Results for cefepime, ceftazidime, imipenem, meropenem, ciprofloxacin, levofloxacin, piperacillin-tazobactam, gentamicin, amikacin, aztreonam, and tobramycin were reported as susceptible, resistant, or intermediate, according to Clinical and Laboratory Standards Institute (CLSI) guidelines (Wayne, Pennsylvania: Clinical and Laboratory Standards Institute, 2021); breakpoints for these antibiotics did not change during the study period. Minimum inhibitory concentration (MIC) determinations for *P. aeruginosa* isolates were performed by broth microdilution (BMD) in cation-adjusted Mueller Hinton broth, according to CLSI standards. We collected the *P. aeruginosa* isolates from these patients, and stored these isolates in 25% glycerol in LB at −80°C. Some paired isolates from the same patients, with intervals of more than 14 days, were also collected to investigate the evolutionary relationship between MDR and QS deficiency. The study was approved by the SGH Review Board (No. 2020-50).

### Definitions

NCFB was diagnosed according to the permanently dilated airways on high-resolution CT imaging, and the etiologic diagnosis was made according to the guidelines (Martínez-García et al., 2007; Goeminne and Dupont, 2010). Chronic *P. aeruginosa* lung infection was defined as at least two times of *P. aeruginosa* isolation from sputum or bronchoalveolar fluid cultures obtained at an interval of 14 days. The density of *P. aeruginosa* in the sputum sample was more than 10^7^ CFU/mL, and in the BALF sample was more than 10^4^ CFU/ml. Infections were considered ventilator-associated if the isolates were obtained during the treatment of invasive mechanical ventilation. Isolates reported as intermediate or resistant were considered nonsusceptible. MDRPA was defined as acquired nonsusceptibility to three or more anti-*Pseudomonal* antimicrobial classes, such as carbapenems, fluoroquinolones, penicillins/cephalosporins, and aminoglycosides (Magiorakos et al., 2012).

### 3OC12-HSL and C4-HSL measurement

3OC12-HSL was measured using a GFP-reporter strain, which contains *mbaR* under *tac* promoter control and a *gfp* transcriptional fusion to −421 bp upstream of the *mbaI* promoter in the pECP61.5 vector background. C4-HSL was measured using a mCherry-reporter strain as previously reported (Ding et al., 2018). Synthetic 3OC12-HSL and C4-HSL (Cayman Chemical, Ann Arbor, MI, USA) were used to generate standard curves.

### Pyocyanin measurement

Pyocyanin was extracted using chloroform and 0.2 mol/L hydrochloric acid–water as previously described, and the absorbance was measured at 520 nm. The value was converted to the concentration of pyocyanin (mg/ml) by multiplying the optical density at 520 nm by 17.072 (Essar et al., 1990).

### Protease activity measurement

Protease of the *P. aeruginosa* cultures was measured by a commercially available Protease Activity Assay (Abcam Inc., Cambridge, MA, USA), which uses fluorescent dye-labeled casein as a general protease substrate. The fluorescence of the protease-catalyzed peptide fragments is measured at Ex/Em = 540/590 nm. The mass spectrometry grade (MSG), chemically stabilized trypsin was used as a general protease control.

### Whole-genome sequencing

Genomic DNA from available clinical isolates was prepared using a QIAamp DNA Kit (QIAGEN, Valencia, CA, USA). DNA libraries were created using the Nextera XT DNA Library Preparation kit (Illumina, San Diego, CA, USA) and sequencing was performed on an Illumina NextSeq 500 platform. Illumina reads were assembled into larger contigs, which were annotated using RAST (Overbeek et al., 2014) and PATRIC (Wattam et al., 2014). Mega 11.0 was used to create a phylogenetic tree in order to determine the relatedness between isolates. The sequences of clinical isolates (NCBI BioProject ID PRJNA837714) were compared to the reference strain *P. aeruginosa* PAO1 (GenBank sequence AE004091). The ratio of nonsynonymous (Ka) to synonymous (Ks) nucleotide substitution rates was calculated to indicate the selective pressures on genes.

### Analysis of efflux pump activity for *lasR* mutant evolution

Based on the results of WGS, three clinical efflux-pump-regulator mutant MDR isolates with complete *lasR* sequence were selected for evolution experiments for *lasR* mutation. To determine the characteristics of MDR in these strains, they were diluted according to CLSI standards, and MICs of ciprofloxacin, ceftazidime, and meropenem were obtained by Etest (bioMérieux, Marcy-l’Etoile, France). The strain of *P. aeruginosa* PAO1 was used as a control. The efflux inhibitor phenyl-arginine-β-naphthylamide (PAβN) (Sigma-Aldrich; 100 mg/L) was added simultaneously for comparison.

To initiate evolution experiments, we inoculated 3 ml of 1% casein broth in 18-mm tubes with the three efflux-pump-regulator mutants. Inocula were 150 μl of an overnight culture grown in LB-Mops broth, which corresponded to about 1 × 10^8^ colony forming units (CFU). Each mutant was inoculated in two tubes. One was added with 100 mg/L PAβN and the other not. Cultures were grown for 24 h and subcultured into fresh medium every 24 h. At the indicated times, culture aliquots were removed for further analysis. To identify *lasR* mutant, we patched 100 individual colonies on skim milk agar [1/4-strength LB broth/4% (wt/vol) skim milk/1.5% (wt/vol) agar]. After about 18 h, the absence of a clear halo around a colony indicated protease dysfunction (Sandoz et al., 2007). Mutations in *lasR* were identified by PCR, and two independently obtained PCR products from each strain were completely sequenced.

### Statistical analysis

GraphPad Prism (version 9; GraphPad Software, San Diego, CA, USA) was used to analyze the data and generate graphs for visualizing the results. Normally distributed quantitative variables were analyzed using *t*-tests, and non-normally distributed variables were analyzed using the Mann–Whitney *U* test. Qualitative variables were analyzed using the Chi-squared test. *p*-values of <0.05 were considered statistically significant.

## Results

### Patients characteristics

A total of 97 patients with chronic *P. aeruginosa* lung infection were involved in the analysis, including 64 men and 33 women with a mean age (± SD) of 66.2 ± 18.8 years. Among these patients, 63 were colonized with non-MDR strains and 34 were colonized with MDR strains. The characteristics of patients colonized with MDR and non-MDR strains are compared in **Table 1**. Univariate analysis showed there was no significant difference in age, gender, etiology, association with a hospital-acquired infection, intensive care unit stay, ventilation-acquired pneumonia, and blood neutrophils between the two groups. However, patients with MDR isolates had a significantly longer duration of infection and higher frequencies of prior carbapenem exposures than those with non-MDR isolates (*p* < 0.05). In the further multivariate analysis, only prior carbapenem exposure (odds ratio 24.9, 95% confidence interval 5.2–156.0, *p* < 0.0001) was found to be independently predictive of the isolation of MDRPA.

**Table 1:** Patient characteristics according to whether they had multidrug-resistant *P. aeruginosa* isolates from respiratory tract samples.

Characteristic	Non-MDR (*n* = 63)	MDR (*n* = 34)	*p-*value
Age (years, median (SD))	64.3 (18.5)	69.6 (19.6)	0.1929
Gender			
Male (*n* (%))	43 (68.3)	21 (61.8)	0.5198
Female (*n* (%))	20 (31.7)	13 (38.2)	
Etiology			
Postinfectious origin	18 (28.6)	11 (32.4)	0.6979
Other origins^a^	14 (22.2)	5 (14.7)	0.3735
Idiopathic^b^	31 (49.2)	18 (52.9)	0.7256
Duration of PA infection (months, median (IQR))	20 (16–38)	54 (21–451)	0.0006
Hospital-associated infection (*n* (%))	41 (65.1)	16 (47.1)	0.0854
ICU stay (*n* (%))	36 (57.1)	14 (41.2)	0.1333
VAP (*n* (%))	29 (46.0)	14 (41.2)	0.6460
Prior antibiotic exposures^c^			
Fluoroquinolone (*n* (%))	9 (14.3)	6 (17.6)	0.6622
Antipseudomonal β-lactam (*n* (%))	8 (12.7)	9 (26.5)	0.0887
Carbapenem (*n* (%))	4 (6.3)	10 (29.4)	0.0020
Blood neutrophils (×10^9^/L, median (SD))	7.3 (4.5)	7.9 (5.1)	0.5948

MDR, multidrug resistance; SD, standard deviation; IQR, interquartile range; ICU, intensive care unit; VAP, ventilator-associated pneumonia.

a Other etiologic diagnoses for bronchiectasis include diffuse panbronchiolitis, allergic bronchopulmonary aspergillosis, connective tissue diseases, immune deficiency, ciliary dyskinesia, and other congenital disorders. These diagnoses were assessed according to clinician criteria, based on the required complementary test results.

b If no compatible etiologic diagnosis was established, bronchiectasis was classified as idiopathic.

c Antibiotic exposures within 90 days of *P. aeruginosa* isolation.

### Deficient QS phenotypes in MDR isolates

**Figure 1:**
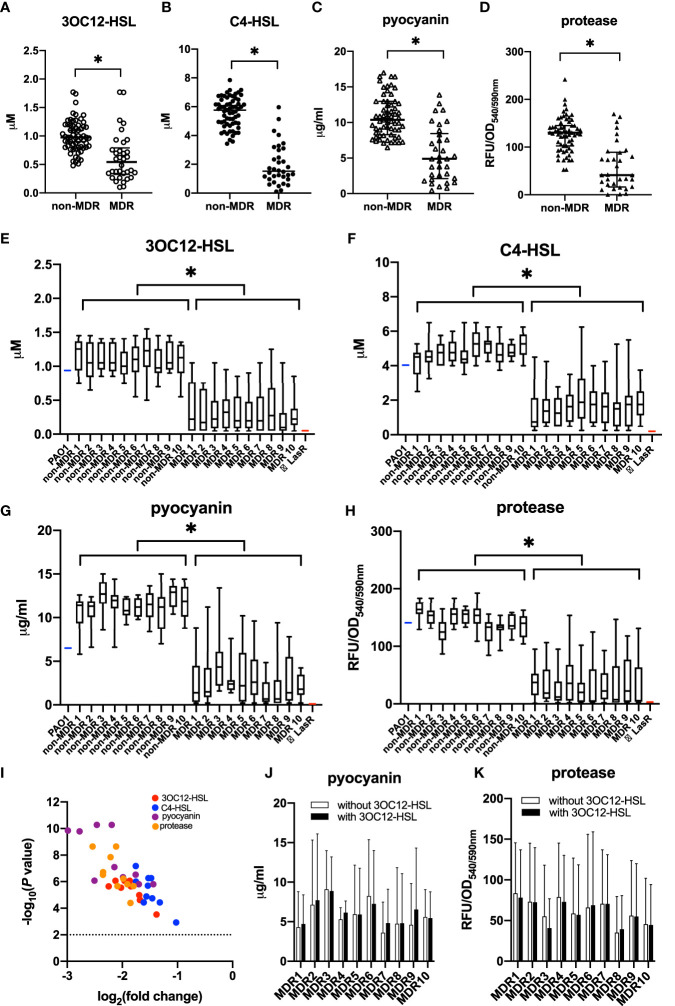
Quorum sensing (QS) phenotypic assessments for *P. aeruginosa* isolates from non-cystic fibrosis bronchiectasis patients. *P. aeruginosa* isolates were classified into the non-MDR group (*n* = 63) and the MDR group (*n* = 34) according to their antimicrobial susceptibility test results. The production of QS signals **(A, B)** and QS-controlled virulence factors **(C, D)** was compared between MDR isolates and non-MDR isolates from different patients. The data were presented as median with an interquartile range. Another 10 strains were isolated within the sample from each of the 20 randomly chosen patients. Among these patients, 10 had MDR isolates, and the others had non-MDR isolates. The production of QS signals **(E, F)** and QS-controlled virulence factors **(G, H)** of MDR isolates or non-MDR isolates within a given patient is shown. PAO1 (blue) and LasR-null mutant (red) served as positive and negative controls, respectively. The whiskers of the boxplot mark the 5th and 95th percentiles, while the box contains the 25th percentile, median, and 75th percentiles. A volcano plot **(I)** was worked out to show the difference of these QS products between non-MDR isolates and MDR isolates. The dotted line presented the significant level of *p* = 0.01. The production of pyocyanin **(J)** and protease **(K)** was compared between MDR cultures with and without 3OC12-HSL from a given patient. The data are presented as mean ± SD. ^*^ *p* < 0.05. MDR, multidrug resistance; OD, optical density; RFU, relative fluorescence unit.

We selected one isolate from a respiratory sample of each patient, and the susceptibility of *P. aeruginosa* isolates was summarized according to whether they were MDR or not (**Table 2**). The QS phenotypes of signal molecules (3OC12-HSL and C4-HSL) and virulent factors (pyocyanin and protease) were compared between MDR and non-MDR isolates (**Figure 1**). We found that MDR isolates had significantly lower production of these QS-controlled signal molecules and virulent factors than non-MDR isolates (*t* = 6.99 for 3OC12-HSL, 14.41 for C4-HSL, 7.72 for pyocyanin, and 7.78 for protease, all *p* < 0.05), and these phenotypes showed more heterogeneity in MDR isolates than in non-MDR isolates (*F =* 2.06 for 3OC12-HSL, 1.87 for C4-HSL, 1.80 for pyocyanin, and 1.97 for protease, all *p* < 0.05, **Figures 1A–D**).

**Table 2:** Susceptibility of *Pseudomonas aeruginosa* isolates according to whether they were multidrug-resistant or not.

Antibiotic	Non-MDR (*n* = 63)	MDR (*n* = 34)	*p*-value
	Insusceptible isolates [*n* (%)]	Insusceptible isolates [*n* (%)]	
Ciprofloxacin	10 (15.9)	23 (67.6)	<0.0001
Levofloxacin	9 (14.3)	21 (61.8)	<0.0001
Meropenem	2 (3.2)	22 (64.7)	<0.0001
Imipenem	3 (4.8)	25 (73.5)	<0.0001
Cefepime	4 (6.3)	24 (70.6)	<0.0001
Ceftazidime	3 (4.8)	20 (58.8)	<0.0001
Gentamicin	7 (11.1)	16 (47.1)	<0.0001
Amikacin	5 (7.9)	18 (52.9)	<0.0001
P/T	1 (1.6)	20 (58.8)	<0.0001
Tobramycin	0 (0)	8 (23.5)	<0.0001

P/T, piperacillin–tazobactam.

To further investigate whether the deficiency and heterogeneity of QS phenotypes existed among MDR strains that colonized the same patient, another 10 strains were isolated within the sample from each of 20 randomly chosen patients. Among these patients, 10 had MDR isolates, and the others had non-MDR isolates. We found that although the MDR isolates from the same sample showed identical resistance to drugs, their QS phenotypes were diversified in the production of signal molecules and virulence factors. As a whole, QS phenotypes were poorer in MDR isolates than in non-MDR isolates throughout all the samples (**Figures 1E–I**), which supported that the emergence of QS deficiency was related to the development of MDR in *P. aeruginosa* during chronic lung infection.

We also measured QS-controlled virulence production of these MDR isolates in the presence or absence of 3OC12-HSL after 18 h of growth and found that their decreased virulence production could not be significantly complemented by the exogenous addition of 3OC12-HSL, suggesting the dysfunction of LasR, rather than LasI, was involved in the deficiency of QS phenotypes in these MDR isolates (**Figures 1J, K** **)**.

**Figure 2:**
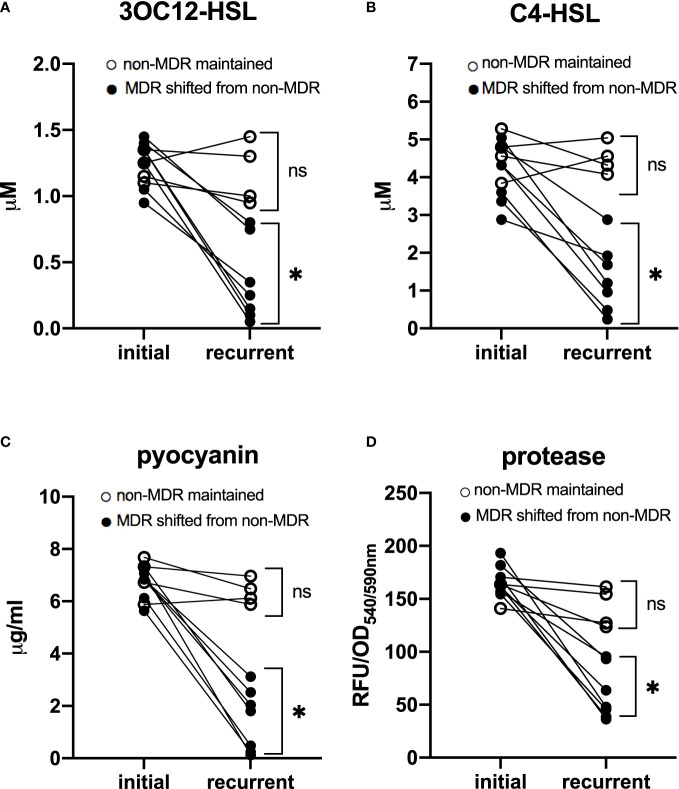
Quorum sensing phenotypes of 11 paired *P. aeruginosa* isolates. Each pair was obtained from a different patient. The time interval between the paired two isolates was 81 (35–98) days apart. In patients who had isolates shifting from non-MDR to MDR, quorum sensing phenotypes, including signal molecules 3OC12-HSL **(A)** and C4-HSL **(B)**, and virulence factors pyocyanin **(C)** and protease **(D)**, were significantly poorer in recurrent isolates than in initial ones; while in patients who had paired isolates maintaining non-MDR, no significant difference of QS phenotypes was found between initial and recurrent isolates. For initial isolates *vs*. recurrent isolates, ^*^ *p* < 0.05; ns, nonsignificant; MDR, multidrug resistance; OD, optical density; RFU, relative fluorescence unit.

In addition, to determine whether there was an evolutionary relationship between the emergence of QS-deficient phenotypes and MDR features, we analyzed 11 paired isolates obtained from different patients. The time interval between the isolates of each pair was 81 (35–98) days apart. Among these patients, seven had paired isolates shifting from non-MDR to MDR, while the other four had paired isolates maintaining non-MDR. We found that in patients who had isolates with MDR shifting, the QS phenotypes of the recurrent isolates were significantly poorer than those of the initial ones (*t* = 6.67 for 3OC12-HSL, 5.96 for C4-HSL, 10.33 for pyocyanin, and 9.97 for protease, *p* < 0.05), while in other patients who had isolates maintaining non-MDR, no significant difference in QS phenotypes was observed between the recurrent and the initial isolates (*t* = 0.28 for 3OC12-HSL, 0.33 for C4-HSL, 1.18 for pyocyanin, and 1.56 for protease, *p* > 0.05, **Figure 2**). These data suggested that QS-deficient phenotypes occurred after the development of MDR in these *P. aeruignosa* strains.

### Identification of MDR determinants and QS gene mutations by WGS

**Figure 3:**
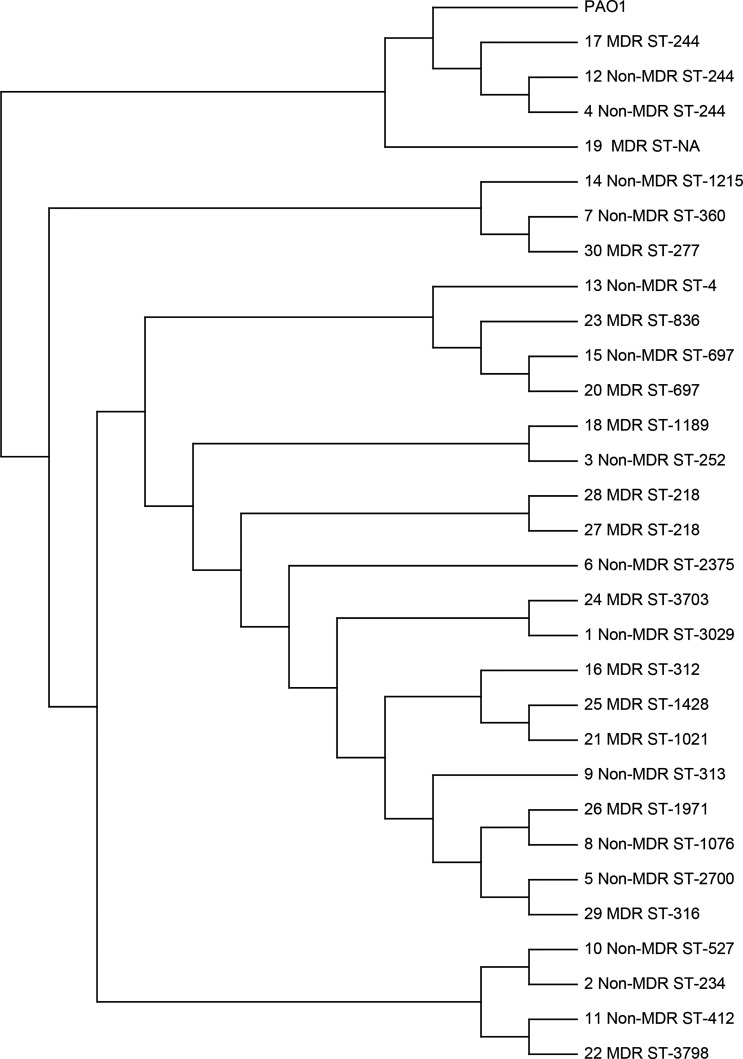
Single-nucleotide variation analysis of *P. aeruginosa* clinical respiratory isolates selected for whole-genome sequencing. The *P. aeruginosa* PAO1 strain (GenBank sequence AE004091) was used as the reference. The paired isolates (15 and 30, 27 and 28, and 23 and 24) were obtained from the same patients.

In order to elucidate the mechanism of QS-deficient phenotypes for MDR isolates in our study population, 30 isolates that had *in vitro* growth rates similar to the laboratory strain PAO1 were chosen to be sent for WGS. These isolates included 24 isolates from different patients and 3 paired isolates from the same patients. A single-nucleotide-variation analysis was performed to determine the degree of genetic relatedness among the sequenced isolates (**Figure 3**). We found that the sequence types were diversified in these isolates, and only a few clusters of closely related isolates were identified, which included ST-244 (*n* = 3), ST-697 (*n* = 2), and ST-218 (*n* = 2). The pair of isolates 27 and 28, both were MDR isolates from one patient obtained 24 days apart, were found to be closely related (ST-218). However, the other two paired isolates from other patients were not closely related in the genetic sequence. The pair of isolates 23 and 24, which were MDR isolates obtained 103 days apart, had clusters of ST-836 and ST-3703, respectively. For the pair of isolates 15 and 30, the initial one was non-MDR and the recurrent one was MDR. The two isolates were obtained 63 days apart and found to have clusters of ST-697 and ST-277, respectively. These data suggested the genetic diversity was high among these sequenced isolates, even for the paired isolates from the same patients.

Mutations in drug resistance determinants were shown in **Table 3**. No isolate was found to harbor a carbapenemase gene. Instead, 14 (93.3%) MDR isolates and 11 (73.3%) non-MDR isolates contained nonsynonymous mutations in *oprD* that were predicted to result in a loss of expression of functional OprD, including disruptive inframe deletion (Lys380_Asn381del), disruptive inframe insertion (Asp374dup, Pro353_Gly354insSer), conservative inframe deletion (Val189del), conservative inframe insertion (Gly383_Tyr384insLeu, Tyr382_Gly383insAla), stop gained (Trp65*, Gln235*), and frameshift variants. However, when we compared the nonsynonymous mutation frequencies of *oprD* between MDR isolates and non-MDR isolates, no significant difference was found between the two groups. Although nonsynonymous mutations existed in other drug resistance determinants, such as *ampC*, *ampR*, *ampD*, and *dacB*, almost all these mutations were missense variants, and neither frameshift nor premature stop codon was found in these genes (**Supplementary Table S1**), suggesting these gene mutations did not play a major role in the MDR development of our isolates.

**Table 3:** Mutations in multidrug resistance determinants, efflux pump regulators, and lasR-I and RhlR-I system.

Isolate	MDR	OprD^a^	MexR	NalC	NalD	MexZ	NfxB	LasR	LasI	RhlR	RhlI
1	N	–	Val126Glu, Ala103Gly	Gly71Glu, Gln182Lys	–	Glu26Lys	His109Tyr	–	–	–	Val148Leu Asp83Glu Ser62Gly
2	N	Val189del, Thr187fs, Pro186fs	–	Gly71Glu	–	–	Thr2Pro	–	–	–	Asp83Glu Ser62Gly
3	N	Gly383_Tyr384insLeu, Asn381fs, Lys380fs, Gly378fs, Val377fs, Asp374dup, Asp374fs, Met372fs, Ser59fs, Ser57fs	–	Gly71Glu	–	–	–	–	–	–	Asp83Glu Ser62Gly
4	N	–	–	–	–	–	–	–	–	–	Asp83Glu Ser62Gly
5	N	Val189del, Thr187fs, Pro186fs	Val126Glu	Gly71Glu, Ala145Val, Ser209Arg	–	–	Arg21His, Asp56Gly	–	–	–	–
6	N	Gly383_Tyr384insLeu, Asn381fs, Lys380fs, Gly378fs, Val377fs, Asp374dup, Asp374fs, Met372fs, Ser59fs, Ser57fs	Val126Glu	Gly71Glu, Ser209Arg	–	Leu138Arg	–	Ser44fs	–	–	Asp83Glu Ser62Gly
7	N	–	Glu109Lys	Gly71Glu, Ser209Arg	–	Leu174Gln	–	–	–	Lys196Arg	Asp83Glu
8	N	Val189del, Thr187fs, Pro186fs	Val126Glu, Leu13_Phe17del	Gly71Glu, Ser209Arg	–	–	–	–	–	–	Asp83GluSer62Gly
9	N	Gly383_Tyr384insLeu, Tyr382_Gly383insAla, Lys380_Asn381del, Gly378fs, Asn376fs, Asp374fs, Met372fs, Val189del, Thr187fs, Pro186fs, Ser59fs, Ser57fs	Val126Glu	Gly71Glu, Ala145Val, Ser209Arg	–	–	–	–	–	–	Asp83GluSer62Gly
10	N	Gly383_Tyr384insLeu, Asn381fs, Lys380fs, Gly378fs, Val377fs, Asp374dup, Asp374fs, Met372fs, Ser59fs, Ser57fs	–	Gly71Glu	Asn130Ser	–	–	–	–	–	Asp83Glu
11	N	Gly383_Tyr384insLeu, Asn381fs, Lys380fs, Gly378fs, Val377fs, Asp374dup, Asp374fs, Met372fs, Ser59fs, Ser57fs	–	Gly71Glu, Ser209Arg	Gly206Ser	–	–	–	–	–	Ala110ValAsp83GluSer62Gly
12	N	–	–	–	–	–	–	–	–	–	Asp83GluSer62Gly
13	N	Gly383_Tyr384insLeu, fAsn381fs, Lys380fs, Gly378fs, Val377fs, Asp374dup Asp374fs, Met372fs, Ser59fs, Ser57fs	Val126Glu	Gly71Glu, Ser209Arg	–	–	–	–	–	–	Asp83GluSer62Gly
14	N	Gly383_Tyr384insLeu,Tyr382_Gly383insAla, Lys380_Asn381del, Gly378fs, Asn376fs, Asp374fs Met372fs, Val189del, Thr187fs, Pro186fs	Gln18fs	Gly71Glu, Ser209Arg	–	–	–	–	–	–	Asp83GluSer62Gly
15	N	Val189del, Thr187fs, Pro186fs, Thr105fs	–	Gly71Glu, Ser209Arg	–	–	–	–	–	–	Asp83GluSer62Gly
16	Y	Gly383_Tyr384insLeu, Tyr382_Gly383insAla, Lys380_Asn381del, Gly378fs, Val377fs, Asp374fs Met372fs, Val189del, Thr187fs, Pro186fs, Gly124fs, Ser59fs, Ser57fs	Val126Glu, Arg23fs	Gly71Glu, Ala145Val, Ser209Arg	–	–	–	–	–	–	–
17	Y	Gln402fs	–	Gly71Glu, Ser209Arg	–	Thr177fs	–	–	–	–	Asp83GluSer62Gly
18	Y	Gln402fs	Lys71fs	Gly71Glu, Ala186Thr	Asp187His	–	–	Ala140Asp	–	–	Asp83Glu
19	Y	Ser59fs, Ser57fs, Trp6X	–	Ser209Arg	–	–	–	–	–	–	Ser62Gly
20	Y	Gly383_Tyr384insLeu, Tyr382_Gly383insAla, Lys380_Asn381del, Gly378fs, Asn376fs, Asp374fs, Met372fs, Val189del, Thr187fs, Pro186fs, Thr105fs	–	Gly71Glu, Ser209Arg	–	Asp209fs	Ala15Thr	–	–	–	Asp83GluSer62Gly
21	Y	Gly212fs, Val189del, Thr187fs, Pro186fs	Val126Glu	Gly71Glu, Ala145Val, Ser209Arg	–	Leu138Arg	Arg21His, Asp56Gly, His87Arg	Gln24X	–	–	–
22	Y	Gln402fs	–	Met19fs, Gly71Glu, Ser209Arg	–	–	–	Ala134_Phe143del	–	–	Asp83GluSer62Gly
23	Y	Gly383_Tyr384insLeu, Asn381fs, Lys380fs, Gly378fs, Val377fs, Asp374dup, Asp374fs, Met372fs, Trp65X, Ser59fs, Ser57fs	Arg23Pro	Gly71Glu, Ser209Arg	–	–	Arg82Leu	–	–	–	Asp83GluSer62Gly
24	Y	Gly383_Tyr384insLeu, Asn381fs, Lys380fs, Gly378fs Val377fs, Asp374dup, Asp374fs, Met372fs, Val189del, Thr187fs, Pro186fs	Val132Met, Val126Glu, Ala103Gly, Ile72Leu	Gly71Glu, Gln182Lys	–	Leu138Arg, Leu196Ile	His109Tyr	–	–	–	Asp83GluSer62Gly
25	Y	Gln235X, Val189del, Thr187fs, Pro186fs	Val126Glu	Gly71Glu, Ala145Val, Ser209Arg	–	Asn186Ser	Arg21His, Asp56Gly	Ala206Val	–	–	–
26	Y	Val189del, Thr187fs, Pro186fs	Val126Glu	Gly71Glu, Glu153Gln, Ser209Arg	Phe132fs	–	–	–	–	–	Asp83GluSer62Gly
27	Y	Gly383_Tyr384insLeu, Asn381fs, Lys380fs, Gly378fs Val377fs, Asp374dup, Asp374fs, Met372fs, Gly354fs Pro353_Gly354insSer, Ser59fs, Ser57fs	–	Gly71Glu, Ser209Arg	–	Val105fs	–	Lys16fs	–	–	Asp83Glu
28	Y	Gly383_Tyr384insLeu, Asn381fs, Lys380fs, Gly378fs, Val377fs, Asp374dup, Asp374fs, Met372fs, Gly354fs, Pro353_Gly354insSer, Ser59fs, Ser57fs	–	Gly71Glu, Ser209Arg	–	Val105fs	–	Lys16fs	–	–	Asp83Glu
29	Y	Gly383_Tyr384insLeu, Tyr382_Gly383insAla, Lys380_Asn381del, Gly378fs, Asn376fs, Asp374fs, Met372fs, Val189del, Thr187fs, Pro186fs, Leu108fs, Ser59fs, Ser57fs	Val126Glu	Gly71Glu, Ser209Arg	start_lost (Met1)?	–	–	–	Ser50Ser	Arg186Ser	Ser62GlyArg22Leu
30	Y	–	–	Gly71Glu, Glu153Gln, Ser209Arg	–	Leu174Gln	–	Asn209fs	–	–	Asp83Glu

a Only inactivating mutations (frameshifts, premature stop codons, inframe deletion, and deletion) are listed.

All mutations are at the amino acid level unless otherwise specified.

del, deletion; fs, frameshift; X, stop codon; ins, insertion; dup, disruptive_inframe_insertion.

We assessed the WGS data of each isolate for mutations in the regulators of efflux pumps MexAB-OprM, MexXY-OprM, and MexCD-OprJ. MexAB-OprM expression is negatively regulated by *mexR*, *nalC*, and *nalD* (**Table 3**). The missense variants of *mexR* (Val126Glu) and *nalC* (Ala145Val, Gly71Glu, Ser209Arg) were common in both MDR and non-MDR isolates. However, the inactivating mutations of *mexR* were different between MDR isolates and non-MDR isolates. The MDR-specific inactivating mutations in *mexR* included frameshift variant Arg23fs (isolate 16) and frameshift variant Lys71fs (isolate 18). For the other two regulators *nalC* and *nalD*, all the inactivating mutations were specific to MDR isolates, including frameshift variant Met19fs (isolate 22), frameshift variant Phe132fs (isolate 26), and start lost Met1? (isolate 29). MexXY-OprM is negatively regulated by *mexZ*, and all of the inactivating mutations in *mexZ* belonged to MDR isolates, including frameshift Thr177fs (isolates 17), Asp209fs (isolate 20), and Val105fs (isolates 27 and 28). For MexCD-OprJ regulator *NfxB*, no inactivating mutations were found in both MDR and non-MDR isolates. As a whole, compared with those in non-MDR isolates, the regulators of MexAB-OprM and MexXY-OprM had higher frequencies of inactivating mutations in MDR isolates (*t* = 7.03, *p* = 0.0080), indicating the involvement of overexpressed efflux pumps in the development of MDR.

Of these 30 sequenced isolates, there were 28 isolates (14 MDR isolates and 14 non-MDR isolates) that had nonsynonymous mutations in *lasR-I* and *rhlR-I* systems coding for polypeptide sequences different from those in the laboratory strain PAO1 (**Table 3**). Among these QS genes, *rhlI* was the most common mutant gene, whose nonsynonymous mutations were present in 26 isolates. However, their mutation frequencies showed no significant difference between MDR isolates and non-MDR isolates (*t* = 1.15, *p* = 0.2827). *lasR* nonsynonymous mutations were present in 8 isolates, and almost all of these isolates belonged to the MDR group. These MDR-specific mutations included stop gained Gln24* (isolate 21), frameshift variant Lys16fs (isolates 27 and 28) and Asn209fs (isolate 30), conservative inframe deletion Ala134_Phe143del (isolate 22), and missense variant Ala140Asp (isolate 18) and Ala206Val (isolate 25). Only a frameshift variant, Ser44fs, in the *lasR* sequence belonged to a non-MDR isolate (isolate 6). *lasI* and *rhlR* mutations were rare, which were only present in two isolates (isolates 7 and 29). These data indicated that *lasR* was the only gene of the *lasR-I* and *rhlR-I* systems that had significantly higher nonsynonymous mutation frequencies in MDR isolates than in non-MDR isolates.

**Figure 4:**
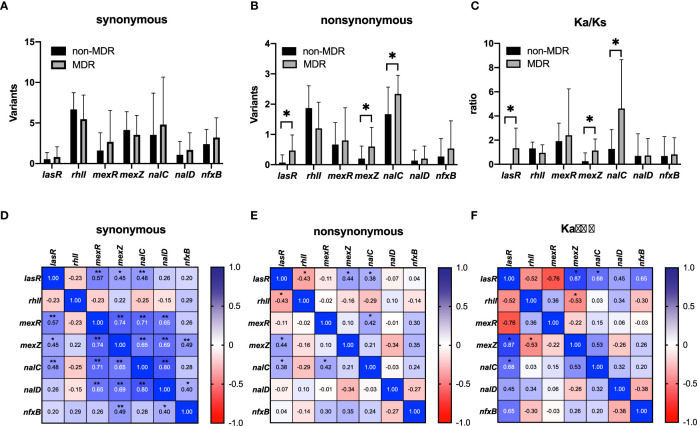
Mutations in quorum sensing genes (*lasR* and *rhlI*) and negative regulator genes (*mexR*, *mexZ*, *nalC*, *nalD*, and *nfxB*) of efflux pumps from non-MDR and MDR *P. aeruginosa* isolates that underwent whole-genome sequencing. A comparison of synonymous mutations **(A)**, nonsynonymous mutations **(B)**, and Ka/Ks ratios **(C)** was analyzed between non-MDR isolates and MDR isolates. Data were presented as mean ± SD. Heatmaps showed the correlation of synonymous mutations **(D)**, nonsynonymous mutations **(E)**, and Ka/Ks ratios **(F)** between non-MDR isolates and MDR isolates. ^*^ *p* < 0.05; ^**^ *p* < 0.01. MDR, multidrug resistance. Ka/Ks ratio is an indicator of selective pressures on genes by calculating the ratio of nonsynonymous (Ka) to synonymous (Ks) nucleotide substitution rates.

The frequencies of synonymous and nonsynonymous mutations in QS genes (*lasR* and *RhlI*) and efflux pump regulator genes (*mexR*, *nalC*, *nalD*, *mexZ*, and *nfxB*) were compared between MDR and non-MDR isolates, and correlations among these frequencies are analyzed in **Figure 4**. We found that there was no significant difference in any synonymous mutation frequency between the two groups (**Figure 4A**); however, nonsynonymous mutation frequencies of *lasR*, *nalC*, and *mexZ* genes were significantly higher in MDR isolates compared with those in non-MDR isolates (**Figure 4B**). Correlation analysis showed that there were significant positive correlations between the synonymous mutation frequencies of *lasR* and efflux pump regulator genes (*mexR*, nal*C*, and *mexZ*) (**Figure 4D**). However, for nonsynonymous mutations, the mutation frequency of *lasR* was found to be only correlated to those of *nalC* and *mexZ* (**Figure 4E**). Furthermore, we calculated the Ka/Ks ratios of these genes to estimate the selection pressure they experienced. We found the Ka/Ks ratios of *lasR*, *nalC*, and *mexZ* were significantly higher in MDR isolates than those in non-MDR isolates (**Figure 4C**), and there were positive correlations between the Ka/Ks ratios of *lasR* and those of *nalC* and *mexZ* (**Figure 4F**), supporting that there was selective pressure for these gene mutations in MDR isolates.

### Phenotypic assessment of efflux pump-induced QS dysfunctionality and evolution experiments

**Figure 5:**
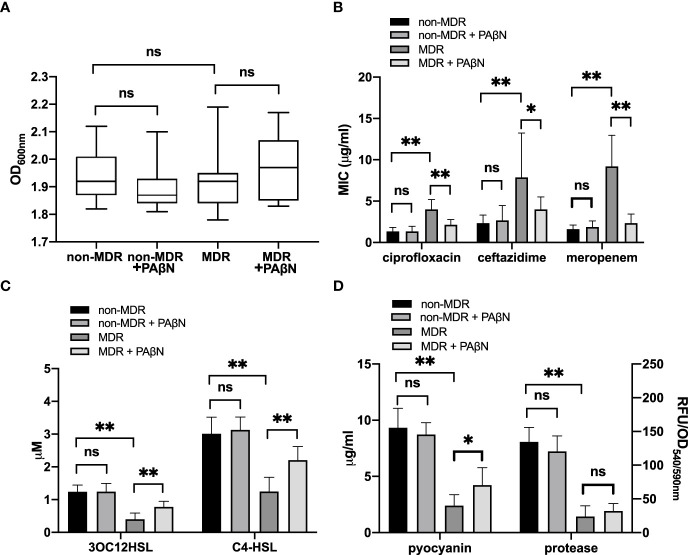
Phenotypic assessment of efflux pump-induced quorum sensing (QS) dysfunctionality. Growth curves showed that bacterial densities were similar among these overnight cultures (18 h) with and without the efflux pump inhibitor PAβN (100 mg/L) **(A)**. MICs of ciprofloxacin, ceftazidime, and meropenem **(B)** and the QS phenotypes, including QS signals **(C)** and virulence factors **(D)**, were analyzed between non-MDR isolates and MDR isolates with or without PAβN. ^*^ *p* < 0.05; ^**^ *p* < 0.01; ns, nonsignificant; MDR, multidrug resistance; MIC, minimum inhibitory concentration; OD, optical density; RFU, relative fluorescence unit.

We used the efflux pump inhibitor PAβN (Sigma-Aldrich; 100 mg/L) to assess the efflux pump activity and its effect on the QS activity of these 30 sequenced isolates. Growth curves showed that bacterial densities were similar among these overnight cultures (18 h) with and without PAβN (**Figure 5A**), which allowed us to compare the antibiotic susceptibility and QS activity for these isolates. We found the addition of PAβN had no significant effect on the MICs of ciprofloxacin, ceftazidime, and meropenem for non-MDR isolates, and the QS phenotypes of these isolates showed no significant difference between cultures with and without PAβN. However, for MDR isolates that had to inactivate mutations in regulators of efflux pumps, the addition of PAβN made the MICs of ciprofloxacin, ceftazidime, and meropenem decrease by more than 50% (**Figure 5B**). Meanwhile, the QS signals and pyocyanin of these MDR isolates were significantly increased by the addition of PAβN (**Figures 5C, D**). These data support efflux pump activity was involved in the development of MDR in these strains, which could result in the low production of QS signal molecules and virulence factors.

To confirm the selective pressure of efflux-pump-controlled factors for *lasR* mutation, we conducted three independent laboratory evolution experiments for each of the three clinical QS-proficient efflux-pump-regulator mutants that had complete *lasR* sequence (isolates 17, 20, and 24). These mutants were identified to be MDR strains by their high MICs of meropenem (MIC ≥ 16 μg/ml for isolates 20 and 24, and 8 μg/ml for isolate 17), ciprofloxacin (MIC ≥ 4 μg/ml for isolates 17 and 20, and 2 μg/ml for isolate 24), and ceftazidime (MIC ≥ 64 μg/ml for isolates 17 and 20, and 32 µg/ml for isolate 24). The strain of *P. aeruginosa* PAO1 was used as a control.

**Figure 6:**
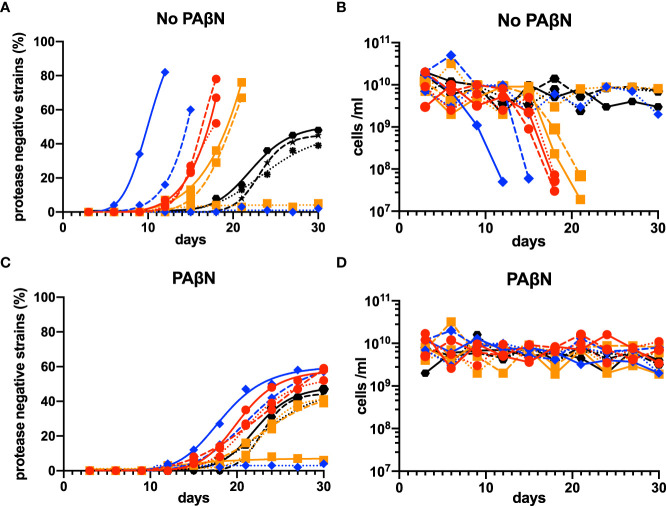
Evolutionary experiments for lasR mutants among MDR *P. aeruginosa* isolates in casein broth medium. Isolates 17, 20, and 24, the three QS-proficient efflux-pump-regulator mutants with a complete *lasR* sequence, were selected. PAO1 was used as a control. Three independent laboratory evolution experiments were conducted, and *lasR* mutants (protease-negative strains) were identified by patching 100 individual colonies on skim milk agar and observing if there was an absence of a clear halo around a colony after about 18 h. The percentage of mutant cells **(A)** and numbers of total cells **(B)** were calculated. These experiments were repeated by adding the efflux pump inhibitor PAβN (100 mg/L) to the casein broth medium **(C, D)**. In all panels, wild-type PAO1 is indicated by black hexagons, the isolate 17 by red circles, the isolate 20 by blue rhombuses, and the isolate 24 by orange squares. The first experiments are shown by the solid lines, the second experiments by the dashed lines, and the third experiments by the dotted lines. MDR, multidrug resistance; QS, quorum sensing.

In the evolutionary experiments, we found that lasR mutants emerged in seven of the nine experiments at variable times. Once they emerged, their relative abundance increased more rapidly than PAO1, and the experiments had to be terminated when there was no growth after the transfer (**Figures 6A, B**). To confirm that the population phenotype was attributable to the upregulation of efflux pump activity, we repeated the experiments by adding the efflux pump inhibitor PAβN (100 mg/L). The addition of PAβN in the casein broth resulted in a stable equilibrium between the QS-proficient and QS-deficient strains in all the repeated experiments and made the phenotypes of MDR strains similar to those of wild-type PAO1 (**Figures 6C, D**).

## Discussion

We performed this study to test the hypothesis that the development of MDR would put selective pressure on the *lasR* mutation in *P. aeruginosa* that colonized NCFB patients. We found that clinical MDR isolates presented with QS-deficient phenotypes, including low production of QS signal molecules and QS-controlled virulence factors. The exogenous addition of signal 3OC12-HSL could not improve these phenotypes. Furthermore, the paired clinical isolates obtained at different times showed that their deficient QS phenotype occurred after MDR was developed. WGS analysis revealed that these clinical MDRPA isolates had upregulated activity of efflux pumps, and there was a positive correlation between the mutation frequencies of *lasR* and efflux pump regulator genes *nalC* and *mexZ*. Evolution experiments supported that upregulated efflux pump activity promoted the emergence of *lasR* mutants among MDR isolates. Our data indicate that MDRPA with upregulated efflux pump activity is susceptible to *lasR* mutation during chronic lung infection.

Some studies showed that therapeutic or prophylactic use of fluoroquinolones, such as ciprofloxacin and levofloxacin, played a major role in the emergence of MDRPA (Paramythiotou et al., 2004; Hakki et al., 2019). However, in our study, no significant difference was found in the frequency of prior fluoroquinolone exposure between patients with and without MDRPA. Instead, prior carbapenem exposure was identified as the independent risk factor for the isolation of MDRPA strains. The difference in antibiotic exposure might be related to empirical antibiotic therapies adopted by physicians according to local epidemiological data (Alnour and Ahmed-Abakur, 2017). For patients with carbapenem exposure, the mechanism of MDR development in colonizing *P. aeruginosa* strains included a lack of OprD protein and upregulation of efflux pump activity (Livermore, 2001). In this study, we found no difference in the nonsynonymous mutation frequencies of *oprD* between non-MDR and MDR strains. However, *nalC* and *mexZ*, the negative regulator genes of efflux pumps MexAB-OprM and MexXY-OprM, had significantly more nonsynonymous mutation frequencies in MDR isolates than in non-MDR isolates, suggesting upregulated activity of efflux pumps, rather than a lack of OprD, was involved in the MDR development of these strains.

Among our clinical isolates, we found evidence of QS deficiency in the MDR isolates tested, and their QS deficiency could not be complemented by the exogenous addition of 3OC12-HSL, suggesting the existence of LasR dysfunction in these MDR isolates. It is worth noting that although QS phenotypes were deficient in our MDR isolates, the diversity of their QS phenotypes was high among these isolates not only from different patients but also within a given patient. The phenomenon could be explained by the evolutionary theory that *lasR* mutants may emerge by social exploitation under certain growth conditions. As *lasR* mutants could benefit from extracellular products made by wild-type without paying the metabolic cost, mixtures of wild-type and mutant cells would form in the populations of *P. aeruginosa* isolates (Wilder et al., 2009). Other factors reported to be involved in QS diversity include coinfection with other microbial species (Harrison, 2007) and heterogeneity of host immune responses (Miller et al., 2005). In this study, as we have excluded patients who had definite coinfections with other microbial species, and no significant difference in blood neutrophil numbers was observed between patients with and without MDR isolates, the impact of microbial coinfections and host immune responses on QS diversity was limited in our isolates. Thus, genetic mutation was considered to be the major factor that contributed to the QS diversity of our MDR isolates, which allowed us to further investigate the genetic makeup of these isolates through WGS analysis.

Our WGS analysis showed that the nonsynonymous mutation frequency of *lasR* was significantly higher in MDR isolates than in non-MDR isolates, which was positively correlated with the frequency of negative regulator genes *nalC* and *mexZ* of efflux pumps. Previous studies reported that upregulated activity of efflux pumps, such as MexAB-oprM, MexCD-OprJ, and MexEF-OprN, was involved in the active efflux of QS signals in *P. aeruginosa* and might alleviate the cost associated with triggering the QS response of neighbor bacteria (Evans et al., 1998; Olivares et al., 2012). MexAB-oprM has been shown to be involved in the active efflux of 3OC12-HSL (Pearson et al., 1999), so it is reasonable to speculate that the upregulated activity of MexAB-oprM caused by *nalC* mutation would lead to low 3OC12-HSL intracellular levels and impair the expression of QS-regulated genes in our MDR strains. When we added efflux pump inhibitor PAβN in the MDR cultures, we saw a significant increase in the production of both QS signals (3OC12-HSL and C4-HSL) and QS-controlled virulence factors (pyocyanin and protease), supporting the detrimental role of upregulated efflux pump activity in the QS function of these MDR isolates. As efflux pump hyperactive mutants, which are defective in producing exoproducts such as proteases for nutrient uptake, could be cheaters supported by neighbor QS-proficient bacteria, the function of LasR was not necessary for their survival. The frequency of *lasR* mutation would be promoted in these strains since the loss of LasR function can reduce their metabolic burden and give them a growth advantage (Sandoz et al., 2007). Our Ka/Ks ratio data supported that there was a positive correlation between selective advantages for the mutation of *lasR* and *nalC* in these MDR isolates. Besides *nalC*, the MexXY-OprM–negative regulator *mexZ* also showed more nonsynonymous mutations in MDR strains, and there was also a positive mutation correlation between *mexZ* and *lasR*. However, the impact of MexXY-OprM on QS signals has not been conclusively documented as other efflux pumps. Direct quantitative analysis of these efflux pumps will be required to determine their contributory role in promoting *lasR* mutation in these isolates.

To confirm the contribution of efflux pumps to *lasR* mutation, we performed the evolution experiments. If the rise of *lasR* mutants was promoted by the upregulated activity of efflux pumps in MDR isolates, the mutant cells should emerge earlier in MDR cultures than those in PAO1 cultures, and the difference between the two groups should be complemented by an efflux pump inhibitor. Consistent with our expectations, we found that in the experiments that had *lasR* mutant rise, the time of mutation emergence was much earlier in the MDR cultures than in PAO1 cultures, and the mutation time could be delayed by the addition of an efflux pump inhibitor. However, to our surprise, the mutants grow so rapidly in the MDR cultures that they overrun the population, causing the population to collapse. A possible reason for the out-of-control mutation was a lack of policing way (e.g., cyanide production) or metabolic constraint (e.g., adenosine metabolism) to restrict mutation emergence in the evolution of MDR isolates (Dandekar et al., 2012; Wang et al., 2015; Yan et al., 2019). More studies are needed to clarify the mechanism involved in the phenomenon.

The development of novel therapies to control lung infection caused by MDR *P. aeruginosa* is one of the major challenges we are currently facing. The QS system poses an important target for anti-infection therapies by regulating genes that are mainly associated with virulence and by avoiding evolutionary pressure on the bacterium to develop resistance (Defoirdt, 2018). However, the inhibitory effect of the upregulated activity of efflux pumps on QS function revealed by our isolates questions the potential therapeutic benefit of using anti-QS therapy in the challenge of MDRPA. A recent study showed some *lasR* mutants still have functional LasR polypeptides with coding variations (Feltner et al., 2016), so targeting the LasR signaling system could still provide therapeutic benefit in the treatment of these MDR strains. In addition, based on our data, efflux pump inhibitors cannot only improve the antibiotic susceptibility but also promote the production of QS signals and delay the occurrence of *lasR* mutants, so the combination of efflux pump inhibitors and anti-QS therapy could be a potential strategy in the challenge of MDRPA infections for NCFB patients (AlMatar et al., 2021).

One limitation of our study was that only two centers were enrolled in the study population, which might limit the generalizability of our findings to the patient populations at other centers, especially if our results were, in part, reflective of the *lasR* mutation in MDR isolates uniquely due to efflux pump hyperactivity. However, as upregulated activity of efflux pumps that can export multiple antibacterial drugs is a common way for *P. aeruginosa* resistant to multiple drugs, it is probable that these upregulated efflux pumps exist in the MDR isolates from other centers and contribute to *lasR* mutation in those isolates. Another point to consider is that we cannot exclude the possibility that other mechanisms were involved in the evolution of *lasR* mutants among MDR isolates, and more studies are needed to clarify them.

Taken together, our study indicated that MDRPA with the upregulated activity of efflux pumps was susceptible to *lasR* mutation during chronic lung infection in NCFB patients. These findings suggest that anti-QS therapy combined with efflux pump inhibitors might be a potential strategy in the challenge of MDR *P. aeruginosa* infections for NCFB patients.

## Data availability statement

The data presented in the study are deposited in the SRA database, accession number PRJNA837714. Release date: 2022-05-13.

## Ethics statement

The studies involving human participants were reviewed and approved by ethics committee of Shanghai General Hospital. The patients/participants provided their written informed consent to participate in this study.

## Author contributions

FD, LH, and MZha conceived of and designed the entire study. YX, IY, XF, and XT contributed to data collection and statistical analyses. YX, LH, FD, PS, and RT performed the experiments. CZ and MZhu performed the bioinformatic analysis. FD and LH wrote the manuscript, which was supervised by MZha. All authors critically reviewed and approved the final version. All authors agreed to be accountable for all aspects of the work in ensuring that questions related to the accuracy or integrity of any part of the work are appropriately investigated and resolved.

## Funding

This work was supported by the National Natural Science Foundation of China (Grant No. 81970006 and Grant No. 81873402); the Project of Science and Technology Commission of Shanghai Municipality (Grant No. 20ZR1444300, Grant No. 20Y11902400, and Grant No. 20Z11900903); the Appropriate Technique Application Program of Shanghai Municipal Health Bureau (Grant No. 2019SY042); and the Three-Year Action Plan of Shanghai Shenkang Hospital Development Center (Grant No. SHDC2020CR5010).

## Acknowledgments

We thank Maxim Kostylev from the Greenberg lab at Washington University, Seattle, WA, USA, and Yue Zheng from the College of the Environment and Ecology, Xiamen University, Fujian, China, for their technical assistance in this work.

## Conflict of interest

CZ and MZhu are employed by Dinfectome Inc., China.

The remaining authors declare that the research was conducted in the absence of any commercial or financial relationships that could be construed as a potential conflict of interest.

## Publisher’s note

All claims expressed in this article are solely those of the authors and do not necessarily represent those of their affiliated organizations, or those of the publisher, the editors and the reviewers. Any product that may be evaluated in this article, or claim that may be made by its manufacturer, is not guaranteed or endorsed by the publisher.
