# Supplementary material for: Multidrug-resistant Pseudomonas aeruginosa is predisposed to lasR mutation through up-regulated activity of efflux pumps in non-cystic fibrosis bronchiectasis patients
Source: Front Cell Infect Microbiol. 2022 Jul 27;12:934439. doi: 10.3389/fcimb.2022.934439 (PMC9363577; doi:10.3389/fcimb.2022.934439)
Supplement: Supplementary file 1 [file Table_1.docx]

Table S1 Mutations in *ampC*, *ampD*, *am*p*R*, and *dacB*.

| Isolate | MDR | ampC | ampD | ampR | dacB |
| --- | --- | --- | --- | --- | --- |
| 1 | N | Arg5Gly, Thr21Ala, Pro23Ser, Ala36Thr, Thr105Ala, Gln117Leu, Ala170Thr, Leu200Ile, Val205Leu, Asp263Asn, Arg273Lys, Val356Ile, Gly391Ala | Glu170Gly, Gly148Ala, Ala85Gly | Gly283Glu | Ala394Pro |
| 2 | N | Thr105Ala, Val205Leu | Gly148Ala | -- | Ala394Pro |
| 3 | N | Thr105Ala | Met1_His2insLeuLeuAlaHisGlnThrProLeuSerArgArgGlyPheSerProLysSerValSerGlyIleSerArgProLeu* |  | Ala394Pro |
| 4 | N |  | Asp183Tyr, Gly148Ala, Arg11Leu | -- | -- |
| 5 | N | Gly27Asp, Thr105Ala, Val205Leu, Gly391Ala |  | Met288Arg, Gly283Glu, Glu114Ala |  |
| 6 | N |  | Asp183Tyr | -- |  |
| 7 | N | Arg79Gln, Thr105Ala | Ser175Leu, Gly148Ala | Gly140Glu | Ala394Pro |
| 8 | N | Gly27Asp, Thr105Ala, Val205Leu, Gly391Ala |  | Met288Arg, Gly283Glu, Glu114Ala |  |
| 9 | N | Pro7Ser, Gly27Asp, Thr105Ala, Val205Leu, Val356Ile, Gly391Ala |  | Met288Arg, Gly283Glu, Ile251Val, Glu114Ala |  |
| 10 | N | Thr105Ala | Asp183Tyr, Gly148Ala | Met288Arg, Gly283Glu |  |
| 11 | N |  | Gly148Ala | -- | Ala394Pro, Ala474Thr |
| 12 | N |  | Asp183Tyr, Gly148Ala, Arg11Leu | -- | -- |
| 13 | N | Pro7Ser, Gly27Asp, Thr105Ala | -- | Met288Arg, Gly283Glu, Glu114Ala |  |
| 14 | N | Arg79Gln, Thr105Ala | Gly148Ala, Gly46Ser | -- | Ala394Pro |
| 15 | N | Arg79Gln, Thr105Ala | Lys165Gln, Gly148Ala | -- | -- |
| 16 | Y | Gly27Asp, Thr105Ala, Gln155Arg, Val205Leu, Val356Ile, Gly391Ala | -- | Met288Arg, Gly283Glu, Glu114Ala |  |
| 17 | Y |  | Asp183Tyr, Gly148Ala, Arg11Leu | -- | Phe438_Ser439delinsCys** |
| 18 | Y | Thr21Ala, Thr105Ala, Val239Ala, Gly391Ala | Pro41Ser, His36Arg | -- |  |
| 19 | Y |  |  | Met288Arg, Gly283Glu | -- |
| 20 | Y | Arg79Gln, Thr105Ala | Gly148Ala | Asp135Gly | -- |
| 21 | Y | Phe19Leu, Thr105Ala, Arg114Ser, Val205Leu, Gly391Ala | Gly148Ala | Asp135Asn |  |
| 22 | Y | Thr105Ala | Gly148Ala | -- | Ala394Pro |
| 23 | Y | Thr105Ala | Gly148Ala, Gly46Ser | -- |  |
| 24 | Y | Thr105Ala, Gly391Ala | Glu170Gly, Gly148Ala, Ala85Gly | Gly283Glu, Glu114Ala | Ala394Pro |
| 25 | Y | Gly27Asp, Thr105Ala, Val205Leu, Val356Ile, Gly391Ala |  | Met288Arg, Gly283Glu |  |
| 26 | Y | Pro23Ser, Thr105Ala, Gly391Ala |  | Met288Ar, Gly283Glu |  |
| 27 | Y | Asp154Asn |  | Met288Arg, Gly283Glu |  |
| 28 | Y | Asp154Asn |  | Met288Arg, Gly283Glu |  |
| 29 | Y | Thr105Ala, Val205Leu, Val356Ile, Gly391Ala |  | Met288Arg, Gly283Glu, Glu114Ala |  |
| 30 | Y | Arg79Gln, Thr105Ala | Ser175Leu, Gly148Ala |  | Ala394Pro |

*conservative_inframe_insertion

**disruptive_inframe_deletion
